# Evaluating Winding Numbers and Counting Complex Roots Through Cauchy Indices in Isabelle/HOL

**DOI:** 10.1007/s10817-019-09521-3

**Published:** 2019-04-03

**Authors:** Wenda Li, Lawrence C. Paulson

**Affiliations:** Computer Laboratory, University of Cambridge, Cambridge, UK

**Keywords:** Interactive theorem proving, Isabelle/HOL, Computer algebra, Cauchy index, Winding number, Root counting, The Routh–Hurwitz stability criterion

## Abstract

In complex analysis, the winding number measures the number of times a path (counter-clockwise) winds around a point, while the Cauchy index can approximate how the path winds. We formalise this approximation in the Isabelle theorem prover, and provide a tactic to evaluate winding numbers through Cauchy indices. By further combining this approximation with the argument principle, we are able to make use of remainder sequences to effectively count the number of complex roots of a polynomial within some domains, such as a rectangular box and a half-plane.

## Introduction

The winding number, given by

$$\begin{aligned} n(\gamma ,z) = \frac{1}{2 \pi i} \oint _\gamma \frac{d w}{w - z}, \end{aligned}$$

measures how the path $\gamma$ winds around the complex point *z*. It is an important object in complex analysis, and its evaluation is ubiquitous among analytic proofs.

However, when formally evaluating the winding number in proof assistants such as Isabelle/HOL and HOL Light, unexpected difficulties arise, as pointed out by Harrison [8] and Li et al. [14]. To address this problem, we formalise a theory of the Cauchy index on the complex plane, thereby approximating how the path winds. When the path is a cycle and comprises line segments and parts of circles, we can now evaluate the winding number by calculating Cauchy indices along those sub-paths.

In addition, by further combining our previous formalisation of the argument principle [14] (which associates the winding number with the number of complex roots), we build effective procedures to count the complex roots of a polynomial within some domains, such as a rectangle box and a half-plane.

In short, the main contributions of this paper are

- a novel tactic to enable users to evaluate the winding number through Cauchy indices,
- and novel verified procedures to count complex roots of a polynomial.

The Isabelle sources of this paper are available from the Archive of Formal Proofs [11, 12].

Formulations in this paper, such as the definition of the Cauchy index and statements of some key lemmas, mainly follow Rahman and Schmeisser’s book [19, Chapter 11] and Eisermann’s paper [6]. Nevertheless, we were still obliged to devise some proofs on our own, as discussed later.

This paper continues as follows: we start with a motivating example (Sect. 2) to explain the difficulty of formal evaluation of the winding number in Isabelle/HOL. We then present an intuitive description of the link between the winding number and the Cauchy indices (Sect. 3), which is then developed formally (Sect. 4). Next, we present verified procedures that count the number of complex roots in a domain (Sect. 5), along with some limitations (Sect. 6) and make some general remarks on the formalisation (Sect. 7). Finally, we discuss related work (Sect. 8) and present conclusions (Sect. 9).

## A Motivating Example

In the formalisation of Cauchy’s residue theorem [14], we demonstrated an application of this theorem to formally evaluate an improper integral in Isabelle/HOL:

1 $$\begin{aligned} \int _{-\infty }^{\infty } \frac{d x}{x^2+1} = \pi . \end{aligned}$$

**Fig. 1:**
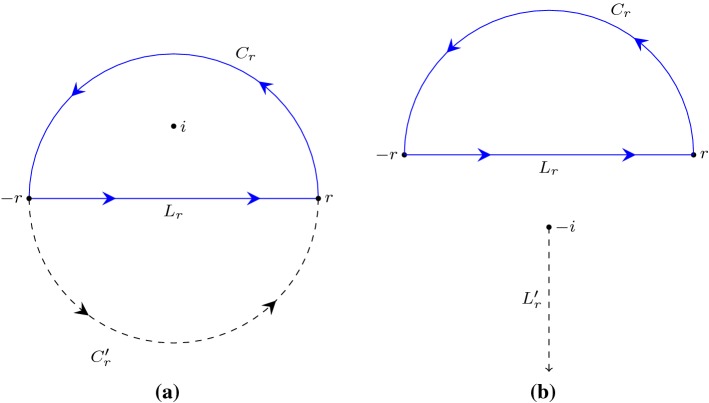
Complex points $(0,-i)$ and (0, *i*), and a closed path $L_r+C_r$

The idea is to embed this integral into the complex plane, and, as illustrated in Fig. 1, to construct a linear path $L_r$ from $-r$ to *r* and a semi-circular path $C_r$ centred at 0 with radius $r>1$:

$$\begin{aligned} C_r (t)= & {} r e^{i \pi t} \quad \mathrm {for}\quad t \in [0,1],\\ L_r (t)= & {} (1-t) (- r) + t r \quad \mathrm {for}\quad t \in [0,1]. \end{aligned}$$

Next, by letting

$$\begin{aligned} f(w) = \frac{1}{w^2 + 1}, \end{aligned}$$

and $r \rightarrow \infty$, we can derive (1) through the following steps:

2 $$\begin{aligned} \int _{-\infty }^{\infty } \frac{d x}{x^2+1}= & {} \oint _{L_r} f \end{aligned}$$

3 $$\begin{aligned}= & {} \oint _{L_r + C_r} f \end{aligned}$$

4 $$\begin{aligned}= & {} n(L_r + C_r,i)\text {Res}(f,i) + n(L_r + C_r,-i)\text {Res}(f,-i) \end{aligned}$$

5 $$\begin{aligned}= & {} \pi . \end{aligned}$$

Here $L_r+C_r$ is formed by appending $C_r$ to the end of $L_r$, and $\text {Res}(f,i)$ is the residue of *f* at *i*. Equation (3) is because $\oint _{C_r} f = 0$ as $r \rightarrow \infty$. The application of the residue theorem is within (4); we exploit the fact that *i* and $-i$ are the only two singularities of *f* over the complex plane, since

$$\begin{aligned} \frac{1}{w^2 + 1} = \frac{1}{(w-i)(w+i)}. \end{aligned}$$

While carrying out the formal proofs of (5), surprisingly, the most troublesome part of the proof is to evaluate the winding numbers:

6 $$\begin{aligned} n(L_r+C_r,i)= & {} 1 \end{aligned}$$

7 $$\begin{aligned} n(L_r+C_r,-i)= & {} 0. \end{aligned}$$

Equations (6) and (7) are straightforward to humans, as it can be seen from Fig. 1 that $L_r+C_r$ passes counterclockwise around the point *i* exactly one time, and around $-i$ zero times. However, formally deriving these facts was non-trivial.

### Example 1

(Proof of $n(L_r+C_r,i) = 1$) We defined an auxiliary semi-circular path $C'_r$ where

$$\begin{aligned} C'_r (t) = r e^{i \pi (t+1)} \quad \hbox {for}\quad t \in [0, 1] \end{aligned}$$

as can be seen in Fig. 1a. As $C_r+C'_r$ forms a (full) circular path with *i* lying inside the circle, we had

8 $$\begin{aligned} n(C_r+C'_r,i) =1. \end{aligned}$$

In addition, we further proved that $C_r+C'_r$ and $L_r+C_r$ are homotopic on the space of the complex plane except for the point *i* (i.e., on ${\mathbb {C}} - \{i\}$), and hence

9 $$\begin{aligned} n(L_r+C_r,i) = n(C_r+C'_r,i) \end{aligned}$$

by using the following Isabelle lemma:

### Lemma 1

(  ) 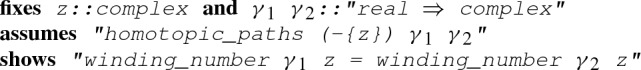


where  encodes the winding number of $\gamma _1$ around *z*: $n(\gamma _1,z)$, and  encodes the homotopic proposition between two paths. Putting (8) and (9) together yields $n(L_r+C_r,i) = 1$, which concludes the whole proof.

### Example 2

(Proof of $n(L_r+C_r,-i) = 0$) We started by defining a ray $L'_r$ starting from $-i$ and pointing towards the negative infinity of the imaginary axis:

$$\begin{aligned} L'_r (t) = (- i) - t i \quad \hbox {for}\quad t \in [0, \infty ) \end{aligned}$$

as illustrated in Fig. 1b. Subsequently, we showed that

10 $$\begin{aligned} L'_R~\text{ does } \text{ not } \text{ intersect } \text{ with }~L_r+C_r, \end{aligned}$$

and then applied the following lemma in Isabelle

### Lemma 2

(  ) 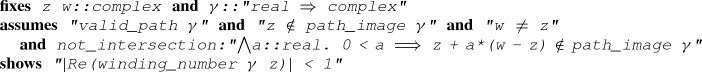


where

assumes that $\gamma$ is piecewise continuously differentiable on [0, 1],
asserts that *z* is not on the path $\gamma$,
- the assumption  asserts that the ray starting at $z \in {\mathbb {C}}$ and through $w \in {\mathbb {C}}$ ($\{z + a (w-z) \mid a>0 \}$) does not intersect with $\gamma$—for all $a>0$, $z + a (w-z)$ does not lie on $\gamma$.

Note that the real part of a winding number $\mathrm {Re}(n(\gamma ,z))$ measures the degree of the winding: in case of $\gamma$ winding around *z* counterclockwise for exactly one turn, we have $n(\gamma ,z) = \mathrm {Re}(n(\gamma ,z)) = 1$. Essentially, Lemma 2 claims that a path $\gamma$ can only wind around *z* for less than one turn, $|\mathrm {Re}(n(\gamma ,z))| < 1$, if there is a ray starting at *z* and not intersecting with $\gamma$. Joining Lemma 2 with (10) leads to

11 $$\begin{aligned} |\mathrm {Re}(n(L_r+C_r,-i))| < 1. \end{aligned}$$

Moreover, as $L_r+C_r$ is a closed path,

12 $$\begin{aligned} n(L_r+C_r,-i) \in {\mathbb {Z}} \end{aligned}$$

By combining (11) and (12), we managed to derive $n(L_r+C_r,-i)=0$.

As can be observed in Examples 1 and 2, our proofs of $n(L_r+C_r,i) = 1$ and $n(L_r+C_r,-i) = 0$ were ad hoc, and involved the manual construction of auxiliary paths or rays (e.g., $C'_R$ and $L'_R$). Similar difficulties have also been mentioned by John Harrison when formalising the prime number theorem [8]. In the next section, we will introduce an idea to systematically evaluate winding numbers.

## The Intuition

The fundamental idea of evaluating a winding number $n(\gamma ,z_0)$ in this paper is to reduce the evaluation to *classifications* of *how* paths cross the line $\{z \mid {\text {Re}}(z)={\text {Re}}(z_0)\}$: continuously or not and in which direction.

**Fig. 2:**
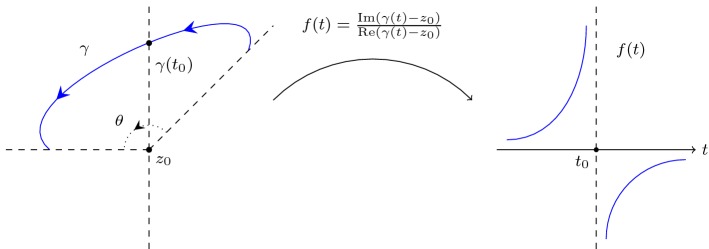
Left: a path $\gamma$ crosses the line $\{z \mid {\text {Re}}(z)={\text {Re}}(z_0)\}$ at $\gamma (t_0)$ such that ${\text {Re}}(\gamma (t_0)) > {\text {Re}}(z_0)$. Right: the image of *f* as a point travels through $\gamma$

In a simple case, suppose a path $\gamma$ crosses the line $\{z \mid {\text {Re}}(z)={\text {Re}}(z_0)\}$ exactly once at the point $\gamma (t_0)$ such that ${\text {Im}}(\gamma (t_0)) > {\text {Im}}(z_0)$ (see Fig. 2 (left)), and let $\theta$ be the change in the argument of a complex point travelling through $\gamma$. It should not be hard to observe that

$$\begin{aligned} 0<\theta < 2 \pi , \end{aligned}$$

and by considering ${\text {Re}}(n(\gamma ,z_0)) = \theta / (2 \pi )$ we can have

$$\begin{aligned} 0< {\text {Re}}(n(\gamma ,z_0)) < 1, \end{aligned}$$

which is an approximation of ${\text {Re}}(n(\gamma ,z_0))$. That is, we have approximated ${\text {Re}}(n(\gamma ,z_0))$ by the way that $\gamma$ crosses the line $\{z \mid {\text {Re}}(z)={\text {Re}}(z_0)\}$.

To make this idea more precise, let

$$\begin{aligned} f(t) = \frac{{\text {Im}}(\gamma (t) - z_0)}{{\text {Re}}(\gamma (t) - z_0)}. \end{aligned}$$

The image of *f* as a point travels through $\gamma$ is as illustrated in Fig. 2 (right), where *f* jumps from $+\infty$ to $-\infty$ across $t_0$. We can then formally characterise those jumps.

### Definition 1

(*Jump*) For $f : {\mathbb {R}} \rightarrow {\mathbb {R}}$ and $x \in {\mathbb {R}}$, we define

$$\begin{aligned} \mathrm {jump}_+(f,x)= & {} {\left\{ \begin{array}{ll} \frac{1}{2} &{} \text{ if } \lim _{u \rightarrow x^+} f(u)=+\infty ,\\ -\frac{1}{2} &{} \text{ if } \lim _{u \rightarrow x^+} f(u)=-\infty ,\\ 0 &{} \text{ otherwise, }\\ \end{array}\right. } \\ \mathrm {jump}_-(f,x)= & {} {\left\{ \begin{array}{ll} \frac{1}{2} &{} \text{ if } \lim _{u \rightarrow x^-} f(u)=+\infty ,\\ -\frac{1}{2} &{} \text{ if } \lim _{u \rightarrow x^-} f(u)=-\infty ,\\ 0 &{} \text{ otherwise. }\\ \end{array}\right. } \end{aligned}$$

Specifically, we can conjecture that $\mathrm {jump}_+(f,t_0) - \mathrm {jump}_-(f,t_0)$ captures the way that $\gamma$ crosses the line $\{z \mid {\text {Re}}(z)={\text {Re}}(z_0)\}$ in Fig. 2, hence ${\text {Re}}(n(\gamma ,z_0))$ can be approximated using $\mathrm {jump}_+$ and $\mathrm {jump}_-$:

$$\begin{aligned} \left| {\text {Re}}(n(\gamma ,z_0)) + \frac{\mathrm {jump}_+(f,t_0) - \mathrm {jump}_-(f,t_0)}{2} \right| < \frac{1}{2}. \end{aligned}$$

In more general cases, we can define Cauchy indices by summing up these jumps over an interval and along a path.

### Definition 2

(*Cauchy index*) For $f : {\mathbb {R}} \rightarrow {\mathbb {R}}$ and $a, b \in {\mathbb {R}}$, the Cauchy index of *f* over a closed interval [*a*, *b*] is defined as

$$\begin{aligned} {\text {Ind}}_a^b(f) = \sum _{x \in [a,b)} \mathrm {jump}_+(f,x) - \sum _{x \in (a,b]} \mathrm {jump}_-(f,x). \end{aligned}$$

### Definition 3

(*Cauchy index along a path*) Given a path $\gamma : [0,1] \rightarrow {\mathbb {C}}$ and a point $z_0 \in {\mathbb {C}}$, the Cauchy index along $\gamma$ about $z_0$ is defined as

$$\begin{aligned} {\text {Indp}}(\gamma ,z_0) = {\text {Ind}}_0^1(f) \end{aligned}$$

where

$$\begin{aligned} f(t) = \frac{{\text {Im}}(\gamma (t) - z_0)}{{\text {Re}}(\gamma (t) - z_0)}. \end{aligned}$$

In particular, it can be checked that the Cauchy index ${\text {Indp}}(\gamma ,z_0)$ captures the way that $\gamma$ crosses the line $\{z \mid {\text {Re}}(z)={\text {Re}}(z_0)\}$, hence leads to an approximation of ${\text {Re}}(n(\gamma ,z_0))$:

$$\begin{aligned} \left| {\text {Re}}(n(\gamma ,z_0)) + \frac{ {\text {Indp}}(\gamma ,z_0) }{2} \right| < \frac{1}{2}. \end{aligned}$$

More interestingly, by further knowing that $\gamma$ is a loop we can derive ${\text {Re}}(n(\gamma ,z_0)) = n(\gamma ,z_0) \in {\mathbb {Z}}$ and ${\text {Indp}}(\gamma ,z_0) / 2 \in {\mathbb {Z}}$, following which we come to the core proposition of this paper:

### Proposition 1

Given a valid path $\gamma : [0,1] \rightarrow {\mathbb {C}}$ and a point $z_0 \in {\mathbb {C}}$, such that $\gamma$ is a loop and $z_0$ is not on the image of $\gamma$, we have

$$\begin{aligned} n(\gamma ,z_0) = - \frac{{\text {Indp}}(\gamma ,z_0)}{2}. \end{aligned}$$

That is, under some assumptions, we can evaluate a winding number through Cauchy indices!

**Fig. 3:**
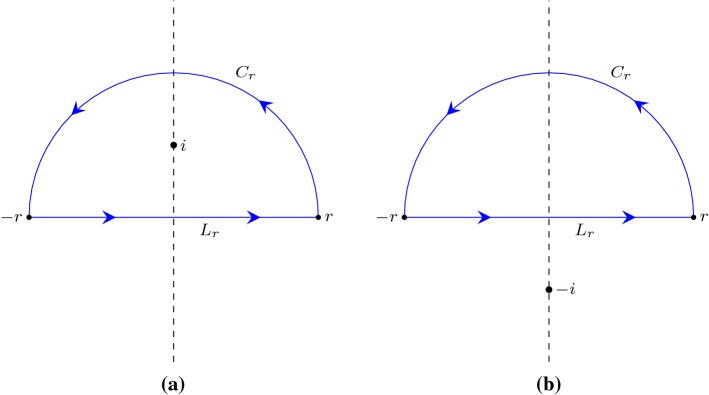
Evaluating $n(L_r+C_r,i)$ and $n(L_r+C_r,-i)$ through the way that the path $L_r+C_r$ crosses the imaginary axis

A formal proof of Proposition 1 will be introduced in Sect. 4.1. Here, given the statement of the proposition, we can have alternative proofs for $n(L_r+C_r,i) = 1$ and $n(L_r+C_r,-i) = 0$.

### Example 3

(Alternative proof of $n(L_r+C_r,i) = 1$) As $L_r+C_r$ is a loop, applying Proposition 1 yields

$$\begin{aligned} n(L_r+C_r,i) = - \frac{{\text {Indp}}(L_r+C_r,i)}{2} = -\frac{1}{2} ({\text {Indp}}(L_r,i) + {\text {Indp}}(C_r,i)), \end{aligned}$$

which reduces $n(L_r+C_r,i)$ to the evaluations of ${\text {Indp}}(L_r,i)$ and ${\text {Indp}}(C_r,i)$. In this case, by definition we can easily decide ${\text {Indp}}(L_r,i) = -1$ and ${\text {Indp}}(C_r,i) = -1$ as illustrated in Fig. 3a. Hence, we have

$$\begin{aligned} n(L_r+C_r,i) = -\frac{1}{2} ((-1) + (-1)) = 1 \end{aligned}$$

and conclude the proof.

### Example 4

(Alternative proof of $n(L_r+C_r,-i) = 0$) As shown in Fig. 3b, we can similarly have

$$\begin{aligned} \begin{aligned} n(L_R+C_R,-i)&= - \frac{{\text {Indp}}(L_r+C_r,-i)}{2} \\&= -\frac{1}{2} ({\text {Indp}}(L_r,-i) + {\text {Indp}}(C_r,-i))\\&= -\frac{1}{2} (1 + (-1)) = 0 \end{aligned} \end{aligned}$$

by which the proof is completed.

Compared to the previous proofs presented in Examples 1 and 2, the alternative proofs in Examples 3 and 4 are systematic and less demanding to devise once we have a formalisation of Proposition  1, which is what we will introduce in the next section.

## Evaluating Winding Numbers

The previous section presented an informal intuition to systematically evaluate winding numbers; in this section, we will report the formal development of this intuition. We will first present a mechanised proof of Proposition 1 (Sect. 4.1), which includes mechanised definitions of jumps and Cauchy indices (i.e., Definition 1, 2 and 3) and several related properties of these objects. After that, we build a tactic in Isabelle/HOL that is used to mechanise proofs presented in Example 3 and 4 (Sect. 4.2). Finally, we discuss some subtleties we encountered during the formalisation (Sect. 4.3).

### A Formal Proof of Proposition 1

For $\mathrm {jump}_-$ and $\mathrm {jump}_+$ (see Definition 1), we have used the filter mechanism [9] to define a function  : 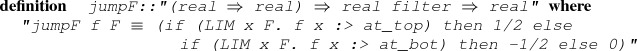 and encoded $\mathrm {jump}_-(f,x)$ and $\mathrm {jump}_+(f,x)$ as

and  ,

respectively. Here,  ,  ,  , and  are all filters, where a filter is a predicate on predicates that satisfies certain properties. Filters are extensively used in the analysis library of Isabelle to encode varieties of logical quantification: for example,  encodes the statement “for a variable that is sufficiently close to *x* from the left", and  represents “for a sufficiently large variable". Furthermore,  encoded the proposition

13 $$\begin{aligned} \lim _{u \rightarrow x^-} f(u) = +\infty , \end{aligned}$$

and this encoding can be justified by the following equality in Isabelle: 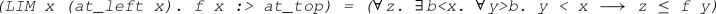 where  matches the usual definition of (13) in textbooks.

We can then encode ${\text {Ind}}_a^b(f)$ and ${\text {Indp}}(\gamma ,z_0)$ (see Definitions 2 and 3) as  and  respectively: 
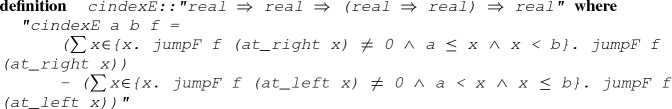

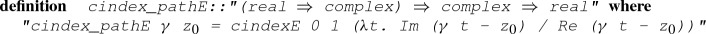
 Note, in the definition of ${\text {Ind}}_a^b(f)$ we have a term

$$\begin{aligned} \sum _{x \in [a,b)} \mathrm {jump}_+(f,x) \end{aligned}$$

which actually hides an assumption: that only a finite number of points within the interval [*a*, *b*) contribute to the sum. This assumption is made explicit when  is defined by summing jumps over the following set: 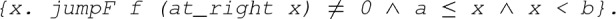 If the set above is infinite (i.e., the sum $\sum _{x \in [a,b)} \mathrm {jump}_+(f,x)$ is not mathematically well-defined) we have 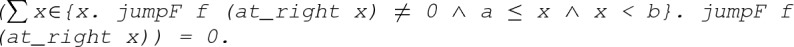 In other words, Isabelle/HOL deems the sum over an infinite set to denote zero.

Due to the issue of well-defined sums, many of our lemmas related to  should assume a finite number of jumps: 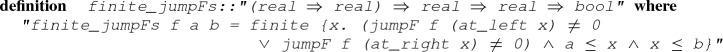 which guarantees the well-definedness of  .

Now, suppose that we know that ${\text {Indp}}$ is well-defined: there are only a finite number of jumps over the path. What strategy can we employ to formally prove Proposition 1? Naturally, we may want to divide the path into a finite number of segments (subpaths) separated by those jumps, and then perform inductions on these segments. To formalise the finiteness of such segments, we defined an inductive predicate: 
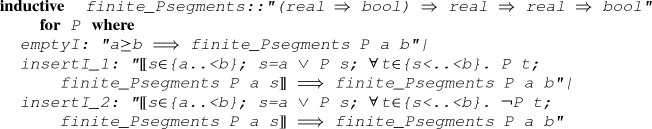

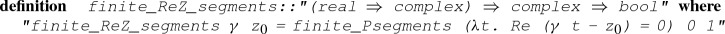
 The idea behind  is that a jump of

$$\begin{aligned} f(t) = \frac{{\text {Im}}(\gamma (t) - z_0)}{{\text {Re}}(\gamma (t) - z_0)} \end{aligned}$$

takes place only if $\lambda t.\, {\text {Re}}(\gamma (t) - z_0)$ changes from 0 to $\ne 0$ (or vice versa). Hence, each of the segments of the path $\gamma$ separated by those jumps has either $\lambda t.\, {\text {Re}}(\gamma (t) - z_0) = 0$ or $\lambda t.\, {\text {Re}}(\gamma (t) - z_0) \ne 0$.

As can be expected, the finiteness of jumps over a path can be derived by the finiteness of segments:

#### Lemma 3

(  ) 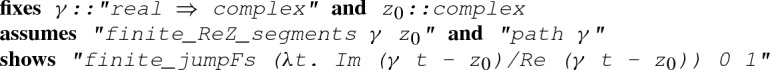


where  asserts that $\gamma$ is a continuous function on [0..1] (so that it is a path). Roughly speaking, Lemma 3 claims that a path will have a finite number of jumps if it can be divided into a finite number of segments.

By assuming such a finite number of segments we have well-defined  , and can then derive some useful related properties:

#### Lemma 4

(  ) 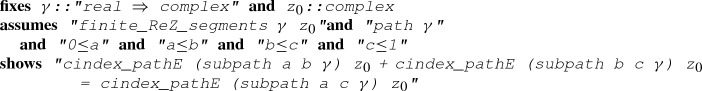


where  gives a sub-path of $\gamma$ based on parameters *a* and *b*: 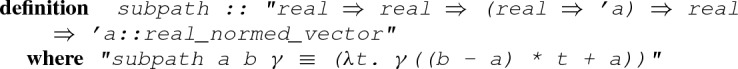


Essentially, Lemma 4 indicates that we can combine Cauchy indices along consecutive parts of a path: given a path $\gamma$ and three parameters *a*, *b*, *c* with $0 \le a \le b \le c \le 1$, we have

$$\begin{aligned} {\text {Indp}}(\gamma _1,z_0) + {\text {Indp}}(\gamma _2,z_0) = {\text {Indp}}(\gamma _3,z_0). \end{aligned}$$

where $$\gamma _1 = \lambda t.\, \gamma ((b-a) t + a)$$, $$\gamma _2 = \lambda t.\, \gamma ((c-b) t + b)$$ and $$\gamma _3 = \lambda t.\, \gamma ((c-a) t + a)$$.

More importantly, we now have an induction rule for a path with a finite number of segments:

#### Lemma 5

(  ) 
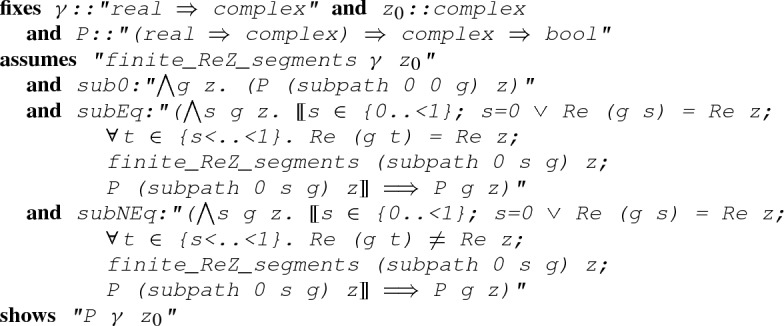


where  is a predicate that takes a path  and a complex point  , and

is the base case that  holds for a constant path;
is the inductive case when the last segment is right on the line $\{x \mid {\text {Re}}(x) = {\text {Re}}(z) \}$: $\forall t \in (s,1).\, {\text {Re}}(g(t)) = {\text {Re}}(z)$;
is the inductive case when the last segment does not cross the line $\{x \mid {\text {Re}}(x) = {\text {Re}}(z) \}$: $\forall t \in (s,1).\, {\text {Re}}(g(t)) \ne {\text {Re}}(z)$.

Given a path $\gamma$ with a finite number of segments, a complex point $z_0$ and a predicate *P* that takes a path and a complex number and returns a boolean, Lemma 5 provides us with an inductive rule to derive $P(\gamma ,z_0)$ by recursively examining the last segment.

**Fig. 4:**
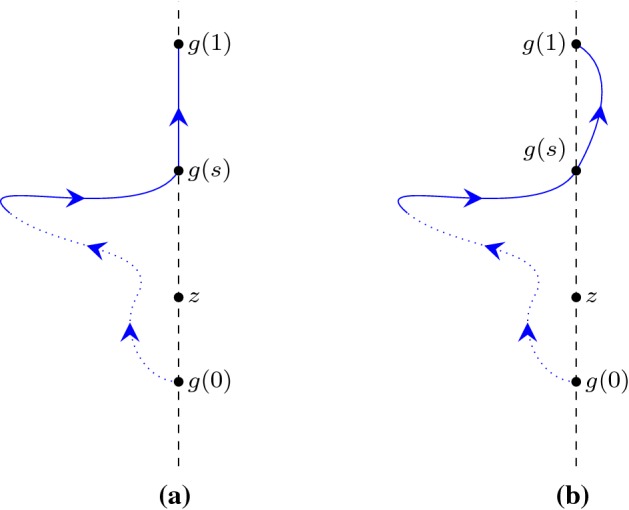
Inductive cases when applying Lemma 5

Before attacking Proposition 1, we can show an auxiliary lemma about ${\text {Re}}(n(\gamma ,z_0))$ and ${\text {Indp}}(\gamma ,z_0)$ when the end points of $\gamma$ are on the line $\{z \mid {\text {Re}}(z) = {\text {Re}}(z_0) \}$:

#### Lemma 6

(  ) 
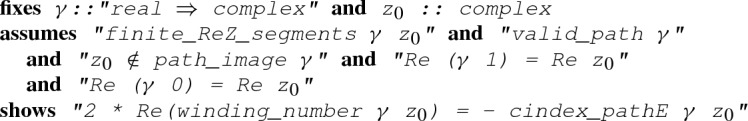


Here, Lemma 6 is almost equivalent to Proposition 1 except for that more restrictions haven been placed on the end points of $\gamma$.

#### Proof of Lemma 6

As there are a finite number of segments along $\gamma$ (i.e.,  ), by inducting on these segments with Lemma 5 we end up with three cases. The base case is straightforward: given a constant path $g : [0,1] \rightarrow {\mathbb {C}}$ and a complex point $z \in {\mathbb {C}}$, we have ${\text {Re}}(n(g,z)) = 0$ and ${\text {Indp}}(g,z) = 0$, hence $2 {\text {Re}}(n(g,z)) = - {\text {Indp}}(g,z)$.

For the inductive case when the last segment is right on the line $\{x \mid {\text {Re}}(x) = {\text {Re}}(z) \}$, there is $\forall t \in (s,1).\, {\text {Re}}(g(t)) = {\text {Re}}(z)$ as illustrated in Fig. 4a. Let

$$\begin{aligned} g_1(t)= & {} g (s t)\\ g_2(t)= & {} g ((1-s) t). \end{aligned}$$

We have

14 $$\begin{aligned} n(g,z) = n(g_1,z) + n(g_2,z), \end{aligned}$$

and, by the induction hypothesis,

15 $$\begin{aligned} 2 {\text {Re}}(n(g_1,z)) = - {\text {Indp}}(g_1,z). \end{aligned}$$

Moreover, it is possible to derive

16 $$\begin{aligned} 2 {\text {Re}}(n(g_2,z)) = - {\text {Indp}}(g_2,z), \end{aligned}$$

since $n(g_2,z) = 0$ and ${\text {Indp}}(g_2,z) = 0$. Furthermore, by Lemma 4 we can sum up the Cauchy index along $g_1$ and $g_2$:

17 $$\begin{aligned} {\text {Indp}}(g_1,z) + {\text {Indp}}(g_2,z) = {\text {Indp}}(g,z) \end{aligned}$$

Combining Eqs. (14), (15), (16) and (17) yields

18 $$\begin{aligned} \begin{aligned} 2 {\text {Re}}(n(g,z))&= 2 ({\text {Re}}(n(g_1,z)) + {\text {Re}}(n(g_2,z))) \\&= - {\text {Indp}}(g_1,z) - {\text {Indp}}(g_2,z)\\&= - {\text {Indp}}(g,z) \end{aligned} \end{aligned}$$

which concludes the case.

For the other inductive case when the last segment does not cross the line $\{x \mid {\text {Re}}(x) = {\text {Re}}(z) \}$, without loss of generality, we assume

19 $$\begin{aligned} \forall t \in (s,1).\, {\text {Re}}(g(t)) > {\text {Re}}(z), \end{aligned}$$

and the shape of *g* is as illustrated in Fig. 4b. Similar to the previous case, by letting $g_1(t) = g (s t)$ and $g_2(t) = g ((1-s) t)$, we have $n(g,z) = n(g_1,z) + n(g_2,z)$ and, by the induction hypothesis, $2 {\text {Re}}(n(g_1,z)) = - {\text {Indp}}(g_1,z)$. Moreover, by observing the shape of $g_2$ we have

20 $$\begin{aligned} 2 {\text {Re}}(n(g_2,z))= & {} \mathrm {jump}_-(f, 1) -\mathrm {jump}_+(f,0) \end{aligned}$$

21 $$\begin{aligned} {\text {Indp}}(g_2,z)= & {} \mathrm {jump}_+(f, 0) - \mathrm {jump}_-(f,1) \end{aligned}$$

where $f(t) = {{\text {Im}}(g_2(t) - z)}/{{\text {Re}}(g_2(t) - z)}$. Combining (20) with (21) leads to $2 {\text {Re}}(n(g_2,z)) = - {\text {Indp}}(g_2,z)$, following which we finish the case by deriving $2 {\text {Re}}(n(g,z)) = - {\text {Indp}}(g,z)$ in a way analogous to (18). $\square$

**Fig. 5:**
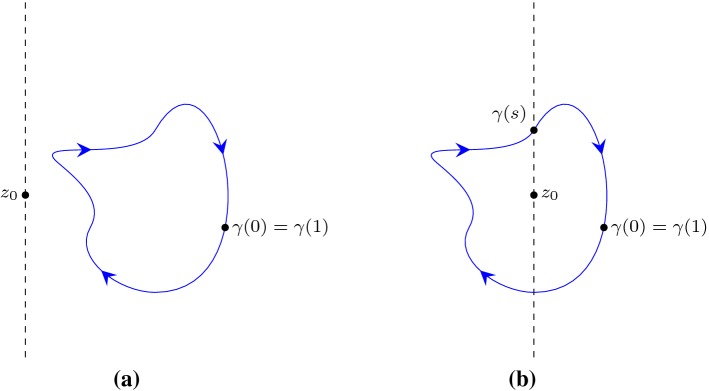
To derive $n(\gamma ,z_0) = - \frac{{\text {Indp}}(\gamma ,z_0)}{2}$ when $\gamma$ is a loop

Finally, we are ready to formally derive Proposition 1 in Isabelle/HOL:

#### Theorem 1

(  ) 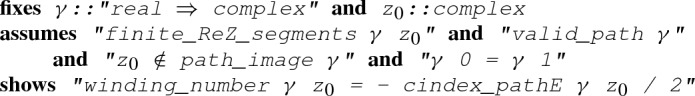


#### Proof

By assumption, we know that $\gamma$ is a loop, and the point $\gamma (0) = \gamma (1)$ can be away from the line $\{z \mid {\text {Re}}(z) = {\text {Re}}(z_0) \}$ which makes Lemma 6 inapplicable. To resolve this problem, we look for a point $\gamma (s)$ on $\gamma$ such that $0 \le s \le 1$ and ${\text {Re}}(\gamma (s)) = {\text {Re}}(z_0)$, and we can either fail or succeed.

In the case of failure, without loss of generality, we can assume ${\text {Re}}(\gamma (t)) > {\text {Re}}(z_0)$ for all $0 \le t \le 1$, and the shape of $\gamma$ is as illustrated in Fig. 5a. As the path $\gamma$ does not cross the line $\{z \mid {\text {Re}}(z) = {\text {Re}}(z_0) \}$, we can evaluate

$$\begin{aligned} {\text {Indp}}(\gamma ,z_0)= & {} 0\\ n(\gamma ,z_0)= & {} {\text {Re}}(n(\gamma ,z_0)) = \frac{{\text {Im}}({\text {Ln}}(\gamma (1) - z_0)) - {\text {Im}}({\text {Ln}}(\gamma (0) - z_0))}{2 \pi } = 0 \end{aligned}$$

where ${\text {Ln}}$ is the principle value of a complex logarithm function with its branch being the negative real axis and $-\pi <{\text {Im}}(Ln(z)) \le \pi$ for all *z*. Hence, $n(\gamma ,z_0) = - {\text {Indp}}(\gamma ,z_0) / 2$ which concludes the case.

In the case of success, as illustrated in Fig. 5b, we have ${\text {Re}}(\gamma (s)) = {\text {Re}}(z_0)$. We then define a shifted path $\gamma _s$:

$$\begin{aligned} \gamma _s(t) = {\left\{ \begin{array}{ll} \gamma (t+s) &{} \text{ if }\quad s+t \le 1,\\ \gamma (t+s-1) &{} \text{ otherwise, }\\ \end{array}\right. } \end{aligned}$$

such that ${\text {Re}}(\gamma _s(0)) = {\text {Re}}(\gamma _s(1)) = {\text {Re}}(z_0)$. By applying Lemma 6, we obtain a relationship between ${\text {Re}}(n(\gamma _s,z_0))$ and ${\text {Indp}}(\gamma _s, z_0)$:

$$\begin{aligned} 2 {\text {Re}}(n(\gamma _s,z_0)) = - {\text {Indp}}(\gamma _s,z_0), \end{aligned}$$

following which we have $n(\gamma ,z_0) = - {\text {Indp}}(\gamma ,z_0) / 2$, since $n(\gamma _s,z_0) = n(\gamma ,z_0)$ and ${\text {Indp}}(\gamma _s,z_0) = {\text {Indp}}(\gamma ,z_0)$. $\square$

### A Tactic for Evaluating Winding Numbers

With Proposition 1 formalised, we are now able to build a tactic to evaluate winding numbers using Cauchy indices. The idea has already been sketched in Examples 3 and 4. We have built a tactic  , for goals of the form

22 $$\begin{aligned} n(\gamma _1+\gamma _2+\cdots +\gamma _n,z_0) = k, \end{aligned}$$

where *k* is an integer and $\gamma _j$ ($1 \le j \le n$) is either a linear path:

$$\begin{aligned} \gamma _j(t) = (1-t) a + t b \quad \text{ where }\quad a, b \in {\mathbb {C}} \end{aligned}$$

or a part of a circular path:

$$\begin{aligned} \gamma _j(t) = z+ r e^{i((1-t) a + t b)} \quad \text{ where }\quad a, b, r \in {\mathbb {R}}\quad \text{ and }\quad z \in {\mathbb {C}}. \end{aligned}$$

The tactic  will transform (22) into

23 $$\begin{aligned}&\gamma _j(1) = \gamma _{j+1}(0)\quad \text{ for } \text{ all }~1 \le j \le n-1,\quad \text{ and }\quad \gamma _n(1) = \gamma _1(0), \end{aligned}$$

24 $$\begin{aligned}&z_0 \not \in \{\gamma _j(t) \mid 0 \le t \le 1 \}\quad \text{ for } \text{ all }~1 \le j \le n, \end{aligned}$$

25 $$\begin{aligned}&{\text {Indp}}(\gamma _1,z_0) + {\text {Indp}}(\gamma _2,z_0) + \cdots + {\text {Indp}}(\gamma _n,z_0) = -2 k, \end{aligned}$$

where (23) ensures that the path $\gamma _1+\gamma _2+\cdots +\gamma _n$ is a loop; (24) certifies that $z_0$ is not on the image of $\gamma _1+\gamma _2+\cdots +\gamma _n$.

To achieve this transformation,  will first perform a substitution step on the left-hand side of Eq. (22) using Theorem 1. As the substitution is conditional, we will need to resolve four extra subgoals (i.e., (26), (27), (28) and (29) as follows) and Eq. (22) is transformed into (30):2627282930To simplify (26), the tactic will keep applying the following introduction rule:^1^

#### Lemma 7

(  ) 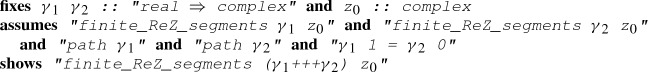


to eliminate the path join operations (  ) until the predicate  is only applied to a linear path or a part of a circular path, and either of these two cases can be directly discharged because these two kinds of paths are proved to be divisible into a finite number of segments by the imaginary axis:

#### Lemma 8

(  ) 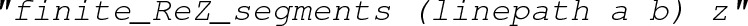


#### Lemma 9

(  ) 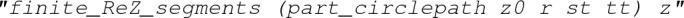


In terms of other subgoals introduced when applying Lemma 7, such as  ,  and  , we can discharge them by the following introduction and simplification rules (all of which have been formally proved):

,
,
,
,
.

As a result,  will eventually simplify the subgoal (26) to (23).

Similar to the process of simplifying (26) to (23), the tactic  will also simplify

- (27) to (23),
- (28) to (24),
- and (29) to (23).

Finally, with respect to (30), we can similarly rewrite with a rule between the Cauchy index (  ) and the path join operation (  ):

#### Lemma 10

(  ) 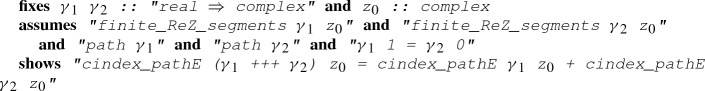


to convert the subgoal (30) to (23) and (25).

After building the tactic  , we are now able to convert a goal like Eq. (22) to (23), (24) and (25). In most cases, discharging (23) and (24) is straightforward. To derive (25), we will need to formally evaluate each ${\text {Indp}}(\gamma _j,z_0)$ ($1 \le j \le n$) when $\gamma _j$ is either a linear path or a part of a circular path.

When $\gamma _j$ is a linear path, the following lemma grants us a way to evaluate ${\text {Indp}}(\gamma _j,z_0)$ through its right-hand side:

#### Lemma 11

(  ) 
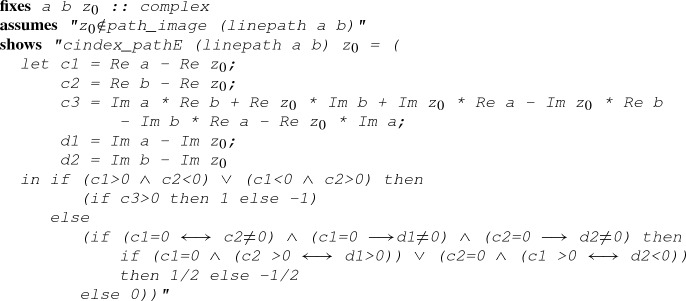


Although Lemma 11 may appear terrifying, evaluating its right-hand side is usually automatic when the number of free variables is small. For example, in a formal proof of Example 3 in Isabelle/HOL, we can have the following fragment: 
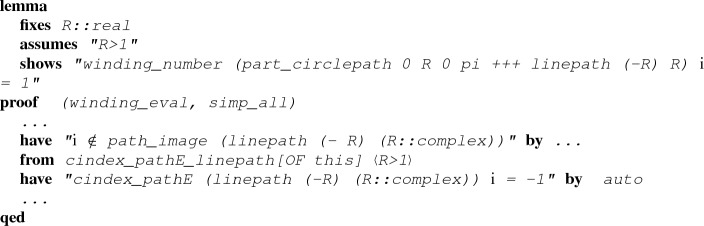
 where  is first applied to convert the goal into (23), (24) and (25), and  subsequently simplifies those newly generated subgoals. In the middle of the proof, we show that the complex point *i* is not on the image of the linear path $L_r$ (i.e.,  in Isabelle/HOL), following which we apply Lemma 11 to derive ${\text {Indp}}(L_r,i) = -1$: the evaluation process is automatic through the command  , given the assumption  .

When $\gamma _j$ is a part of a circular path, a similar lemma has been provided to facilitate the evaluation of ${\text {Indp}}(\gamma _j,z_0)$.

### Subtleties

The first subtlety we have encountered during the formalisation of Proposition 1 is about the definitions of jumps and Cauchy indices, for which our first attempt followed the standard definitions in textbooks [2, 16, 19].

#### Definition 4

(*Jump*) For $f : {\mathbb {R}} \rightarrow {\mathbb {R}}$ and $x \in {\mathbb {R}}$, we define

$$\begin{aligned} \mathrm {jump}(f,x) = {\left\{ \begin{array}{ll} 1 &{} \text{ if } \lim _{u \rightarrow x^-} f(u)=- \infty \text{ and } \lim _{u \rightarrow x^+} f(u)=+\infty ,\\ -1&{} \text{ if } \lim _{u \rightarrow x^-} f(u)=+\infty \text{ and } \lim _{u \rightarrow x^+} f(u)=-\infty ,\\ 0 &{} \text{ otherwise. }\\ \end{array}\right. } \end{aligned}$$

#### Definition 5

(*Cauchy index*) For $f : {\mathbb {R}} \rightarrow {\mathbb {R}}$ and $a, b \in {\mathbb {R}}$, the Cauchy index of *f* over an open interval (*a*, *b*) is defined as

$$\begin{aligned} {\text {Ind}}_a^b(f) = \sum _{x \in (a,b)} \mathrm {jump}(f,x). \end{aligned}$$

**Fig. 6:**
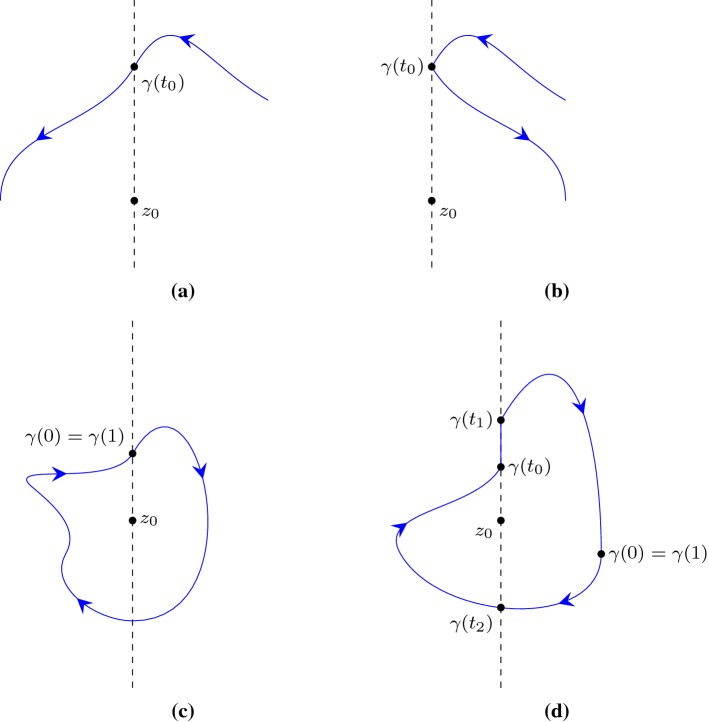
Different ways a path $\gamma$ can intersect with the line $\{z \mid {\text {Re}}(z) = {\text {Re}}(z_0)\}$

The impact of the difference between the current definition of the Cauchy index (i.e., Definition 2) and the classic one (i.e., Definition 5) is small when formalising the Sturm–Tarski theorem [10, 13], where *f* is a rational function. In this case, the path $\gamma$ intersects with the line $\{z \mid {\text {Re}}(z) = {\text {Re}}(z_0)\}$ a finite number of times, and for each intersection point (see Fig. 6a, b), by letting $f(t) = {{\text {Im}}(\gamma (t) - z_0)}/{{\text {Re}}(\gamma (t) - z_0)}$, we have

$$\begin{aligned} \mathrm {jump}(f,t) = \mathrm {jump}_+(f,t) - \mathrm {jump}_-(f,t), \end{aligned}$$

hence

$$\begin{aligned} \sum _{x \in (a,b)} \mathrm {jump}(f,x) = \sum _{x \in [a,b)} \mathrm {jump}_+(f,x) - \sum _{x \in (a,b]} \mathrm {jump}_-(f,x), \end{aligned}$$

provided $\mathrm {jump}_+(f,a) =0$ and $\mathrm {jump}_-(f,b) = 0$. That is, the classic Cauchy index and the current one are equal when *f* is a rational function and does not jump at both ends of the target interval.

Naturally, the disadvantages of Definition 5 are twofold:

- The function $\lambda t.\, {\text {Re}}(\gamma (t) - z_0)$ cannot vanish at either end of the interval. That is, we need to additionally assume ${\text {Re}}(\gamma (0) - z_0) \ne 0$ as in Rahman and Schmeisser’s formulation [19, Lemma 11.1.1 and Theorem 11.1.3], and Proposition 1 will be inapplicable in the case of Fig. 6c where ${\text {Re}}(\gamma (0)) = {\text {Re}}(\gamma (1)) = {\text {Re}}(z_0)$.
- The function $\lambda t.\, {{\text {Im}}(\gamma (t) - z_0)}/{{\text {Re}}(\gamma (t) - z_0)}$ has to be rational, which makes Proposition 1 inapplicable for cases like in Fig. 6d (if we follow Definition 5). To elaborate, it can be observed in Fig. 6d that $n(\gamma ,z_0) = -1$, while we will only get a wrong answer by following Definition 5 and evaluating via Proposition 1:
  $$\begin{aligned} - \frac{1}{2} \left( \sum _{x \in (0,1)} \mathrm {jump}(f,x) \right) = - \frac{\mathrm {jump}(f,t_2)}{2} = - \frac{1}{2}, \end{aligned}$$
  where $f(t) = {{\text {Im}}(\gamma (t) - z_0)}/{{\text {Re}}(\gamma (t) - z_0)}$. In comparison, Definition 2 leads to the correct answer:
  $$\begin{aligned} \begin{aligned} n(\gamma ,z_0)&= - \frac{1}{2} \left( \sum _{x \in [0,1)} \mathrm {jump}_+(f,x) - \sum _{x \in (0,1]} \mathrm {jump}_-(f,x) \right) \\&= - \frac{1}{2} \left( \mathrm {jump}_+(f,t_2) + \mathrm {jump}_+(f,t_1) - \mathrm {jump}_-(f,t_2) - \mathrm {jump}_-(f,t_0) \right) \\&= - \frac{1}{2} \left( \frac{1}{2} + \frac{1}{2} - (- \frac{1}{2}) - (- \frac{1}{2}) \right) \\&= - 1. \end{aligned} \end{aligned}$$

Fortunately, Eisermann [6] recently proposed a new formulation of the Cauchy index that overcomes those two disadvantages, and this new formulation is what we have followed (in Definitions 1 and 2).

Another subtlety we ran into was the well-definedness of the Cauchy index. Such well-definedness is usually not an issue and left implicit in the literature, because, in most cases, the Cauchy index is only defined on rational functions, where only finitely many points can contribute to the sum. When attempting to formally derive Proposition 1, we realised that this assumption needed to be made explicit, since the path $\gamma$ can be flexible enough to allow the function $f(t) = {{\text {Im}}(\gamma (t) - z_0)}/{{\text {Re}}(\gamma (t) - z_0)}$ to be non-rational (e.g., Fig. 6d). In our first attempt of following Definition 5, the Cauchy index was formally defined as follows: 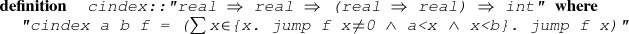 and its well-definedness was ensured by the finite number of times that $\gamma$ crosses the line $\{z \mid {\text {Re}}(z) = {\text {Re}}(z_0) \}$: 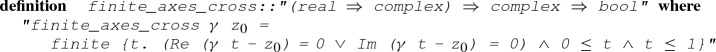 where the part  ensures that  is non-zero only at finitely many points over the interval [0, 1]. When constrained by  , the function $f(t) = {{\text {Im}}(\gamma (t) - z_0)}/{{\text {Re}}(\gamma (t) - z_0)}$ behaves like a rational function. More importantly, the path $\gamma$, in this case, can be divided into a finite number of ordered segments delimited by those points over [0, 1], which makes an inductive proof of Proposition 1 possible. However, after abandoning our first attempt and switching to Definition 2, the well-definedness of the Cauchy index is assured by the finite number of $\mathrm {jump}_+$ and $\mathrm {jump}_-$ of *f* (i.e., Definition  in Sect. 4.1), with which we did not know how to divide the path $\gamma$ into segments and carry out an inductive proof. It took us some time to properly define the assumption of a finite number of segments (i.e., Definition  ) that implied the well-definedness using Lemma 3 and provided a lemma for inductive proofs (i.e., Lemma 5).

## Counting the Number of Complex Roots

The previous section described a way to evaluate winding numbers via Cauchy indices. In this section, we will further explore this idea and propose verified procedures to count the number of complex roots of a polynomial in some domain such as a rectangle and a half-plane.

Does a winding number have anything to do with the number of roots of a polynomial? The answer is yes. Thanks to the argument principle, we can calculate the number of roots by evaluating a contour integral:

31 $$\begin{aligned} \frac{1}{2 \pi i} \oint _\gamma \frac{p'(x)}{p(x) } dx = N \end{aligned}$$

where $p \in {\mathbb {C}}[x]$, $p'(x)$ is the first derivative of *p* and *N* is the number of complex roots of *p* (counted with multiplicity) inside the loop $\gamma$. Also, by the definition of winding numbers, we have

32 $$\begin{aligned} n(p \circ \gamma ,0) = \frac{1}{2 \pi i} \oint _\gamma \frac{p'(x)}{p(x) } d x. \end{aligned}$$

Combining Eqs. (31) and (32) gives us the relationship between a winding number and the number of roots of a polynomial:

33 $$\begin{aligned} n(p \circ \gamma ,0) = N. \end{aligned}$$

And the question becomes: can we evaluate $n(p \circ \gamma ,0)$ via Cauchy indices?

### Roots in a Rectangle

**Fig. 7:**
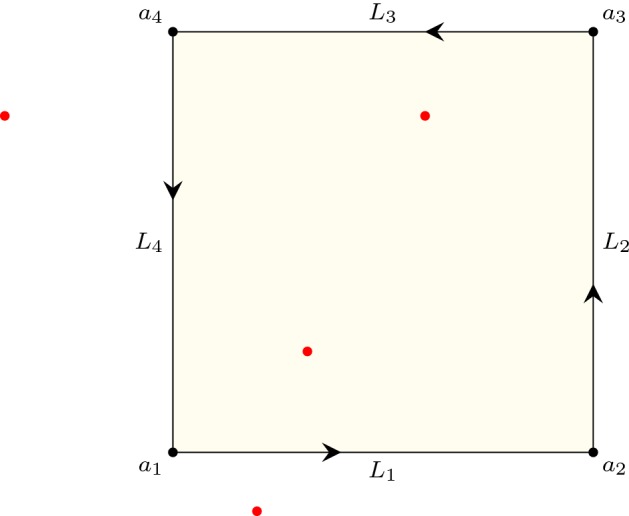
Complex roots of a polynomial (red dots) and a rectangular path ($L_1+L_2+L_3+L_4$) on the complex plane. (Color figure online)

Let *N* be the number of complex roots of a polynomial *p* inside the rectangle defined by its lower left corner $a_1$ and upper right corner $a_3$. As illustrated in Fig. 7, we can define four linear paths along the edge of the rectangle:

$$\begin{aligned} \begin{aligned} L_1(t)&=(1-t) a_1 + t a_2\\ L_2(t)&=(1-t) a_2 + t a_3\\ L_3(t)&=(1-t) a_3 + t a_4\\ L_4(t)&=(1-t) a_4 + t a_1\\ \end{aligned} \end{aligned}$$

where $a_2 = {\text {Re}}(a_3) + i {\text {Im}}(a_1)$ and $a_4 = {\text {Re}}(a_1) + i {\text {Im}}(a_3)$. Combining Proposition 1 with Eq. (33) yields

34 $$\begin{aligned} \begin{aligned} N&= n(p \circ (L_1+L_2+L_3+L_4),0) \\&= -\frac{1}{2} {\text {Indp}}(p \circ (L_1+L_2+L_3+L_4),0) \\&= -\frac{1}{2} \left( {\text {Indp}}(p \circ L_1,0)+ {\text {Indp}}(p \circ L_2,0)+ {\text {Indp}}(p \circ L_3,0) + {\text {Indp}}(p \circ L_4,0)\right) .\\ \end{aligned} \end{aligned}$$

Here, the path $p \circ L_j : [0,1] \rightarrow {\mathbb {C}}$ ($1 \le j \le 4$) is (mostly) neither a linear path nor a part of a circular path, which indicates that the evaluation strategies of Sect. 4.2, such as Lemma 11, will no longer apply. Thankfully, the Sturm–Tarski theorem [10, 13] came to our rescue.

In general, the Sturm–Tarski theorem is about calculating Tarski queries through sign variations and signed remainder sequences: let $p,q \in {\mathbb {R}}[x]$, *a* and *b* be two extended real numbers such that $a<b$ and are not roots of *p*, we have

35 $$\begin{aligned} \mathrm {TaQ}(q,p,a,b) = \mathrm {Var}(\mathrm {SRemS}(p,p' q);a,b) \end{aligned}$$

where

- $p'$ is the first derivative of *p*,
- the Tarski query $\mathrm {TaQ}(q,p,a,b)$ defined as follows:
  $$\begin{aligned} \mathrm {TaQ}(q,p,a,b) = \sum _{x \in (a,b), p(x) = 0} \mathrm {sgn}(q(x)), \end{aligned}$$
- $\mathrm {SRemS}(p,q)$ is the signed remainder sequence started with *p* and *q*.
- Let $[p_1,p_2,\ldots ,p_n]$ be a sequence of polynomials, $\mathrm {Var}([p_1,p_2,\ldots ,p_n];a,b)$ is the difference in the number of sign variations when evaluating $[p_1,p_2,\ldots ,p_n]$ at *a* and *b*:
  36 $$\begin{aligned}&\mathrm {Var}([p_1,p_2,\ldots ,p_n];a,b) \nonumber \\&= \mathrm {Var}([p_1(a),p_2(a),\ldots ,p_n(a)]) - \mathrm {Var}([p_1(b),p_2(b),\ldots ,p_n(b)]). \end{aligned}$$

Note that when $q=1$, (35) becomes the famous Sturm’s theorem, which counts the number of distinct real roots over an interval. For example, by calculating

$$\begin{aligned} \begin{aligned} \mathrm {TaQ}(1,(x-1)(x-2),0,3)&= \mathrm {Var}(\mathrm {SRemS}(x^2-3x+2,2 x - 3);0,3) \\&= \mathrm {Var}([x^2-3x+2,2 x - 3,1/4];0,3) \\&= \mathrm {Var}([x^2-3x+2,2 x - 3,1/4];0) \\&\qquad - \mathrm {Var}([x^2-3x+2,2 x - 3,1/4];3) \\&= \mathrm {Var}([2,-3,1/4]) - \mathrm {Var}([2,3,1/4]) \\&= 2 - 0 = 2, \end{aligned} \end{aligned}$$

we know that the polynomial $x^2-3x+2$ has two distinct real roots within the interval (0, 3).

In our previous formal proof of the Sturm–Tarski theorem [10, 13], we used the Cauchy index to relate the Tarski query and the right-hand side of (35). Therefore, as a byproduct, we can also evaluate the Cauchy index through sign variations and signed remainder sequences:

37 $$\begin{aligned} {\text {Ind}}_a^b \left( \lambda t.\, \frac{q(t)}{p(t)} \right) = \mathrm {Var}(\mathrm {SRemS}(p,q);a,b), \end{aligned}$$

where $p,q \in {\mathbb {R}}[x]$, *a*, *b* are two extended real numbers such that $a<b$ and are not roots of *p*.

Back to the case of ${\text {Indp}}(p \circ L_j,0)$, we have

$$\begin{aligned} {\text {Indp}}(p \circ L_j,0) = {\text {Ind}}_0^1\left( \lambda t.\ \frac{{\text {Im}}(p(L_j(t)))}{{\text {Re}}(p(L_j(t)))} \right) , \end{aligned}$$

and both ${\text {Im}}(p(L_j(t)))$ and ${\text {Re}}(p(L_j(t)))$ happen to be polynomials with real coefficients. Therefore, combining Eqs. (34) and (37) yields an approach to count the number of roots inside a rectangle.

While proceeding to the formal development, the first problem we encountered was that the Cauchy index in Eq. (37) actually follows the classic definition (i.e., Definition 5), and is different from the one in Eq. (34) (i.e., Definitions 2 and 3). Subtle differences between these two formulations have already been discussed in Sect. 4.3. Luckily, Eisermann [6] has also described an alternative sign variation operator so that our current definition of the Cauchy index (i.e., Definition 2) can be computationally evaluated:

#### Lemma 12

(  ) 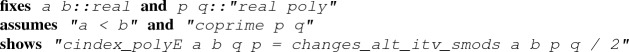


Here,  encodes our current definition of the Cauchy index ${\text {Ind}}^b_a(\lambda t.\, q(t) / p(t))$, and  stands for

38 $$\begin{aligned} {\widehat{\mathrm {Var}}}(\mathrm {SRemS}(p,q);a,b) \end{aligned}$$

where the alternative sign variation operator ${\widehat{\mathrm {Var}}}$ is defined as follows:

$$\begin{aligned} \begin{aligned} {\widehat{\mathrm {Var}}}([p_1,p_2,\ldots ,p_3];a,b)&= {\widehat{\mathrm {Var}}}([p_1,p_2,\ldots ,p_3];a) - {\widehat{\mathrm {Var}}}([p_1,p_2,\ldots ,p_3];b), \\ {\widehat{\mathrm {Var}}}([p_1,p_2,\ldots ,p_3];a)&= {\widehat{\mathrm {Var}}}([p_1(a),p_2(a),\ldots ,p_3(a)]), \\ {\widehat{\mathrm {Var}}}([])&= 0,\\ {\widehat{\mathrm {Var}}}([x_1])&= 0,\\ {\widehat{\mathrm {Var}}}([x_1,x_2,\ldots ,x_n])&= |\mathrm {sgn}(x_1) - \mathrm {sgn}(x_2)| + {\widehat{\mathrm {Var}}}([x_2,\ldots ,x_n]).\\ \end{aligned} \end{aligned}$$

The difference between ${\widehat{\mathrm {Var}}}$ and $\mathrm {Var}$ is that $\mathrm {Var}$ discards zeros before calculating variations while ${\widehat{\mathrm {Var}}}$ takes zeros into consideration. For example, $\mathrm {Var}([1,0,-2]) = \mathrm {Var}([1,-2]) = 1$, while ${\widehat{\mathrm {Var}}}([1,0,-2]) = 2$.

Before implementing Eq. (34), we need to realise that there is a restriction in our strategy: roots are not allowed on the border (i.e., the image of the path $L_1+L_2+L_3+L_4$). To computationally check this restriction, the following function is defined 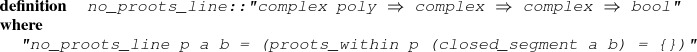 which will return “true” if there is no root on the closed segment between  and  , and “false” otherwise. Here,  is defined as the set $\{(1-u) a + u b \mid 0 \le u \le 1 \} \subseteq {\mathbb {C}}$, and the function  gives the set of roots of the polynomial  within the set  : 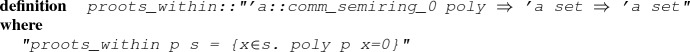


The next step is to make the definition  executable. This is achieved by proving a *code equation*, where the left-hand side of the equation is the target definition and the right-hand side is an executable expression. In the case of  , the code equation is the following lemma:

#### Lemma 13

(  ) 
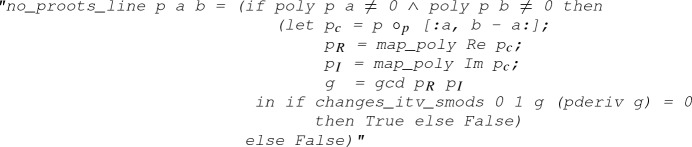


where  is the polynomial composition operation and  and  , respectively, extract the real and imaginary parts of the complex polynomial  .

#### Proof of Lemma 13

Supposing $$L : [0,1] \rightarrow {\mathbb {C}}$$ is a linear path from *a* to *b*: $$ L(t) = (1-t) a + t b$$, we know that $$p \circ L$$ is still a polynomial with complex coefficients. Subsequently, we extract the real and imaginary parts ($$p_R$$ and $$p_I$$, respectively) of $$p \circ L$$ such that

$$\begin{aligned} p (L (t)) = p_R(t) + i p_I(t). \end{aligned}$$

If there is a root of *p* lying right on *L*, we will be able to obtain some $t_0 \in [0,1]$ such that

$$\begin{aligned} p_R(t_0) = p_I(t_0) = 0, \end{aligned}$$

hence, by letting $g=\gcd (p_R,p_I)$ we have $g(t_0) = 0$. Therefore, the polynomial *p* has no (complex) root on *L* if and only if *g* has no (real) root within the interval [0, 1], and the latter can be computationally checked using Sturm’s theorem. $\square$

Finally, we define the function  that returns the number of complex roots of a polynomial (counted with multiplicity) within a rectangle defined by its lower left and upper right corner: 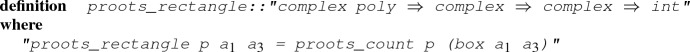 where  denotes the number of roots of the polynomial *p* within the set *s*: 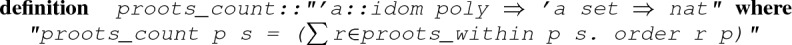 The executability of the function  can be established with the following code equation:

#### Lemma 14

(  ) 
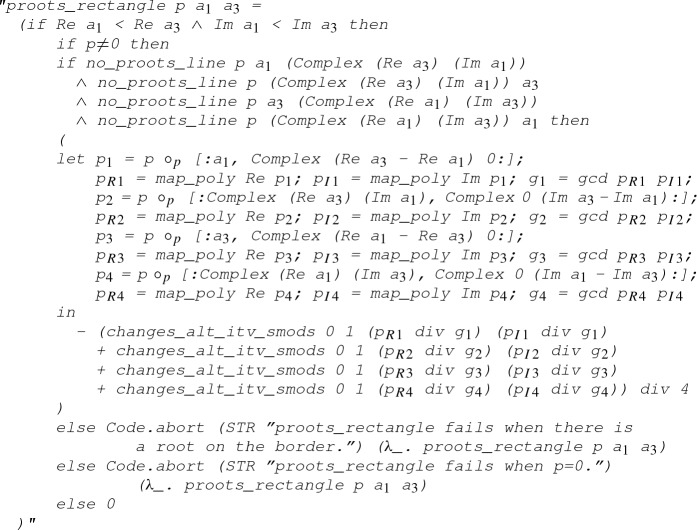


The proof of the above code equation roughly follows Eqs. (34) and (37), where  checks if there is a root of  on the rectangle’s border. Note that the gcd calculations here, such as  , are due to the coprime assumption in Lemma 12.

**Fig. 8:**
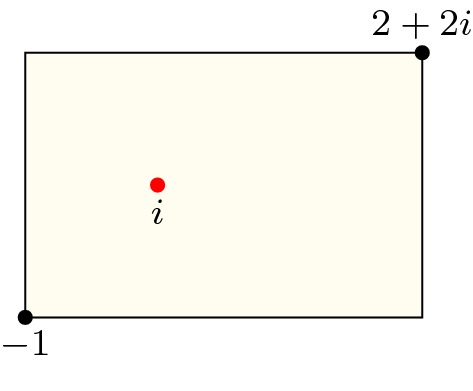
A complex point *i* and a rectangle defined by its lower left corner $-1$ and upper right corner $2+2 i$

#### Example 5

Given a rectangle defined by $(-1, 2 + 2 i)$ (as illustrated in Fig. 8) and a polynomial *p* with complex coefficients:

$$\begin{aligned} p(x) = x^2 - 2 i x - 1 = (x-i)^2 \end{aligned}$$

we can now type the following command to count the number of roots within the rectangle: 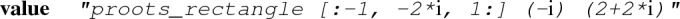 which will return 2 as *p* has exactly two complex roots (i.e., *i* with multiplicity 2) in the area.

### Roots in a Half-plane

**Fig. 9:**
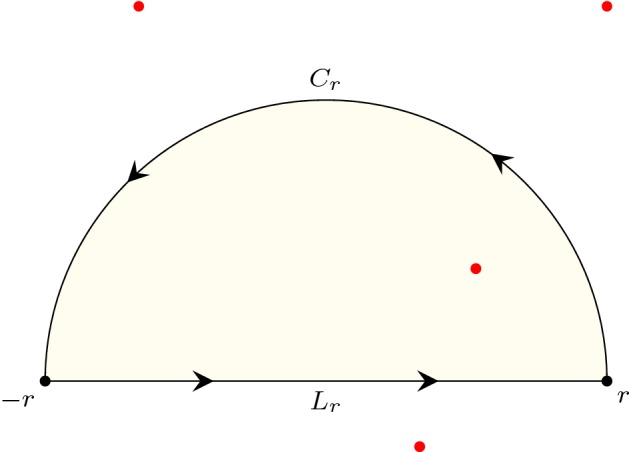
Complex roots of a polynomial (red dots) and a linear path ($L_r$) concatenated by a semi-circular path ($C_r$) on the complex plane. (Color figure online)

For roots in a half-plane, we can start with a simplified case, where we count the number of roots of a polynomial in the upper half-plane of ${\mathbb {C}}$: 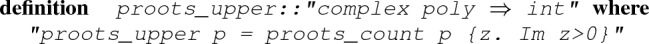 As usual, our next step is to set up the executability of  . To achieve that, we first define a linear path $L_r (t) = (1-t) (- r) + t r$ and a semi-circular path $C_r (t) = r e^{i \pi t}$, as illustrated in Fig. 9. Subsequently, let

$$\begin{aligned} \begin{aligned} C_p(r)&= p \circ C_r \\ L_p(r)&= p \circ L_r, \end{aligned} \end{aligned}$$

and by following Eq. (33) we have

39 $$\begin{aligned} \begin{aligned} N_r&= n(p \circ (L_r+C_r),0) \\&= {\text {Re}}(n(L_p(r), 0)) + {\text {Re}}(n(C_p(r), 0)) \end{aligned} \end{aligned}$$

where $N_r$ is the number of roots of *p* inside the path $L_r+C_r$. Note that as *r* approaches positive infinity, $N_r$ will be the roots on the upper half-plane (i.e.,  ), which is what we are aiming for. For this reason, it is natural for us to examine two cases:

$$\begin{aligned}&\lim _{r \rightarrow +\infty } {\text {Re}}(n(L_p(r), 0)) =\ ?\\&\lim _{r \rightarrow +\infty } {\text {Re}}(n(C_p(r), 0)) =\ ?. \end{aligned}$$

For the case of $\lim _{r \rightarrow +\infty } {\text {Re}}(n(L_p(r), 0))$, we can have

#### Lemma 15

(  ) 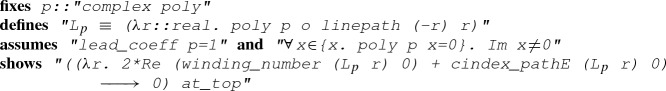


which essentially indicates

40 $$\begin{aligned} \lim _{r \rightarrow +\infty } {\text {Re}}(n(L_p(r), 0)) = - \frac{1}{2} \lim _{r \rightarrow +\infty } {\text {Indp}}(L_p(r),0), \end{aligned}$$

provided that the polynomial *p* is monic and does not have any root on the real axis.

Next, for $\lim _{r \rightarrow +\infty } {\text {Re}}(n(C_p(r), 0))$, we first derive a lemma about $C_r$:

#### Lemma 16

(  ) 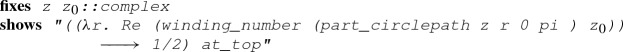


that is, $\lim _{r \rightarrow +\infty } {\text {Re}}(n(C_r, 0)) = 1/2$, following which and by induction we have

#### Lemma 17

(  ) 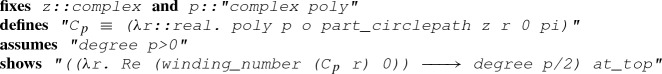


which is equivalent to

41 $$\begin{aligned} \lim _{r \rightarrow +\infty } {\text {Re}}(n(C_p(r), 0)) = \frac{\deg (p)}{2}, \end{aligned}$$

provided $$\deg (p) > 0$$.

Putting Eqs. (40) and (41) together yields the core lemma about  in this section:

#### Lemma 18

(  ) 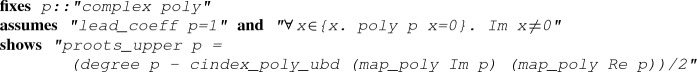


where  is mathematically interpreted as ${\text {Ind}}_{-\infty }^{+\infty } (\lambda t.\, {\text {Im}}(p(t)) / {\text {Re}}(p(t)))$, which is derived from $\lim _{r \rightarrow \infty } {\text {Indp}}(L_p(r),0)$ in Eq. (40) since

$$\begin{aligned} \begin{aligned} \lim _{r \rightarrow +\infty } {\text {Indp}}(L_p(r),0)&= \lim _{r \rightarrow +\infty } {\text {Indp}}(L_p(r),0) \\&= \lim _{r \rightarrow +\infty } {\text {Ind}}_0^1 \left( \lambda t.\ \frac{{\text {Im}}(L_p(r,t))}{{\text {Re}}(L_p(r,t))} \right) \\&= \lim _{r \rightarrow +\infty } {\text {Ind}}_{-r}^r \left( \lambda t.\ \frac{{\text {Im}}(p(t))}{{\text {Re}}(p(t))} \right) \\&= {\text {Ind}}_{-\infty }^{+\infty } \left( \lambda t.\ \frac{{\text {Im}}(p(t))}{{\text {Re}}(p(t))} \right) . \\ \end{aligned} \end{aligned}$$

Finally, following Lemma 18, the executability of the function  is established:

#### Lemma 19

(  ) 
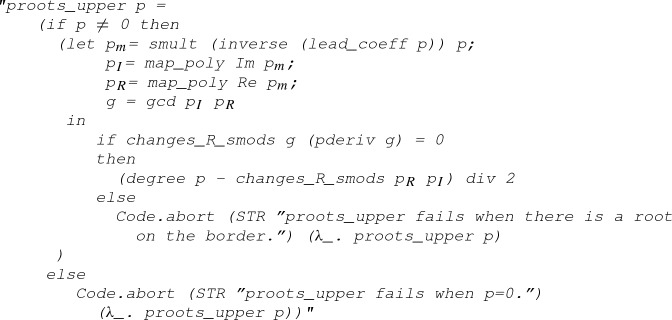


where

divides the polynomial  by its leading coefficient so that the resulting polynomial  is monic. This corresponds to the assumption  in Lemma 18.
checks if  has no root lying on the real axis, which is due to the second assumption in Lemma 18.
evaluates
  $$\begin{aligned} {\text {Ind}}_{-\infty }^{+\infty } \left( \lambda t.\, \frac{{\text {Im}}(p_I(t))}{{\text {Re}}(p_R(t))} \right) \end{aligned}$$
  by following Eq. (37).

As for the general case of a half-plane, we can have a definition as follows: 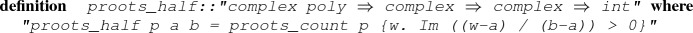 which encodes the number of roots in the left half-plane of the vector $b-a$. Roots of  in this half-plane can be transformed to roots of  in the upper half-plane of ${\mathbb {C}}$:

#### Lemma 20

(  ) 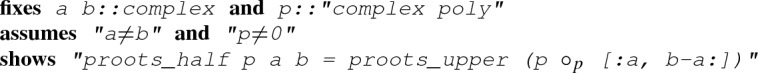


And so we can naturally evaluate  through  :

#### Lemma 21

(  ) 
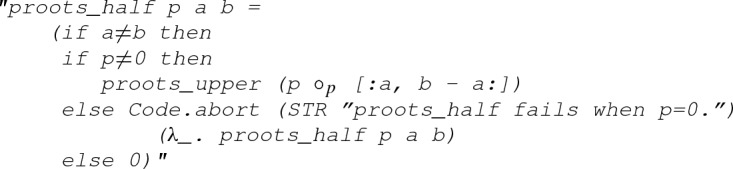


**Fig. 10:**
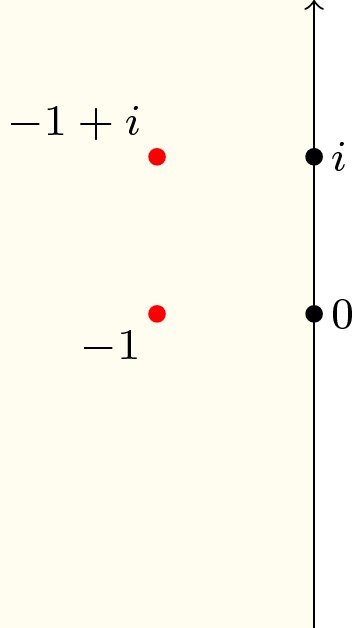
Complex roots of a polynomial (red dots) and a vector (0, *i*). (Color figure online)

#### Example 6

We can now use the following command 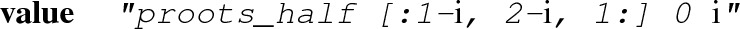 to decide that the polynomial

$$\begin{aligned} p(x) = x^2 + (2-i) x + (1-i) = (x+1) (x + 1 -i) \end{aligned}$$

has exactly two roots within the left half-plane of the vector (0, *i*), as shown in Fig. 10.

Despite our naive implementation, both  and  are applicable for small or medium examples. For most polynomials with coefficient bitsize up to 10 and degree up to 30, our complex root counting procedures terminate within minutes.

## Limitations and Future Work

There are, of course, several improvements that can be made on both the evaluation tactic of Sect. 4.2 and the root counting procedures of Sect. 5. As the tactic is intended to be applied to winding numbers with variables, full automation with this tactic is unlikely in most cases, but we can always aim for better automation and an enhanced interactive experience for users (e.g., presenting unsolved goals in a more user-friendly way).

Regarding the two root-counting procedures in Sect. 5, a key limitation is that they do not allow cases where any root is on the border. There are two possible solutions to this problem:

- To generalise the definition of winding numbers. The current formulation of winding numbers in Isabelle/HOL follows the one in complex analysis:
  $$\begin{aligned} n(\gamma ,z) = \frac{1}{2 \pi i} \oint _\gamma \frac{d w}{w - z} \end{aligned}$$
  which becomes undefined when the point *z* is on the image of the path $\gamma$. With more general formulations of winding numbers, such as the algebraic version by Eisermann [6], we may be able to derive a stronger version of the argument principle that allows zeros on the border.
- To deploy a more sophisticated strategy to count the number of times that the path winds. Recall that the underlying idea in this paper is to reduce the evaluation of winding numbers to *classifications* of how paths cross some line. The Cauchy index merely provides one classification strategy, which we considered simple and elegant enough for formalisation. In contrast, Collins and Krandick [4] propose a much more sophisticated strategy for such classifications. Their strategy has, in fact, been widely implemented in modern systems, such as Mathematica and SymPy, to count the number of complex roots.

Neither of these two solutions are straightforward to incorporate, hence we leave them for future investigation.

Besides rectangles and half-planes, it is also possible to similarly count the number of roots in an open disk and even a sector:

$$\begin{aligned} \mathrm {sector}~(z_0,\alpha ,\beta ) = \{z \mid \alpha< \arg (z - z_0) <\beta \} \end{aligned}$$

where $\arg (-)$ returns the argument of a complex number. Informal proofs of root counting in these domains can be found in Rahman and Schmeisser [19, Chapter 11].

## Potential Applications

Rahman and Schmeisser’s book [19, Chapter 11] and Eisermann’s paper [6] are the two main sources that our development is built upon. Nevertheless, there are still some differences in formulations:

- Rahman and Schmeisser formulated the Cauchy index as in Definitions 4 and 5, and we used their formulation in our first attempt. However, after we realised the subtleties discussed in Sect. 4.3, we abandoned this formulation and switched to Eisermann’s (i.e., Definition 2). As a result, the root counting procedures presented in this paper are more general than the ones in their book, having fewer preconditions.
- Eisermann formulated a winding number $n(\gamma ,z_0)$ in a real-algebraical sense where $\gamma$ is required to be a piecewise polynomial path (i.e., each piece from the path needs to be a polynomial). In comparison, $n(\gamma ,z_0)$ in Isabelle/HOL follows the classic definition in complex analysis, and places fewer restrictions on the shape of $\gamma$ (i.e., piecewise continuously differentiable is less restrictive than being a piecewise polynomial) but does not permit $z_0$ to be on the image of $\gamma$ (while Eisermann’s formulation does). Consequently, Eisermann’s root counting procedure works in more restrictive domains (i.e., he only described the rectangle case in his paper) but does not prevent roots on the border.

Another point worth mentioning is the difference between informal and formal proofs. In this development, we generally treated their lemma statements as bald facts: we had to discover our own proofs. For instance, when proving Proposition 1, we defined an inductive data type for segments and derived an induction rule for it, which was nothing like the informal proof. Such situations also happened when we justified the root counting procedure in a half-plane. Overall, the formal proofs are about 12,000 lines.

Interestingly, the root-counting procedure in a half-plane is also related to the stability problems in the theory of dynamical systems. For instance, let $A \in {\mathbb {R}}^{n \times n}$ be a square matrix with real coefficients and $y: [0,+\infty ) \rightarrow {\mathbb {R}}^n$ be a function that models the system state over time. A linear dynamical system can be described as an ordinary differential equation:

42 $$\begin{aligned} \frac{d y(t)}{d t} = A y(t) \end{aligned}$$

with an initial condition $y(0)=y_0$. The system of (42) is considered stable if all roots of the characteristic polynomial of *A* lie within the open left half-plane (i.e., $\{z \mid {\text {Re}}(z) < 0 \}$), and this stability test is usually referred as the Routh–Hurwitz stability criterion [1, Section 23], [16, Chapter 9]. As has been demonstrated in Example 6, counting the number of roots in the left half-plane is within the scope of the procedure  . For this reason, we believe that the development in this paper will be beneficial for reasoning about dynamical systems in Isabelle/HOL.

It is worth mentioning that root counting in a rectangle is usually coupled with a classic problem in computer algebra, namely, complex root isolation. The basic idea is to keep bisecting a rectangle (vertically or horizontally) into smaller ones until a sub-rectangle contains exactly one root or none (provided the target polynomial is square-free). Following this idea, it is possible to build a simple and verified procedure for complex root isolation similar to Wilf’s algorithm [20]: we start with a large rectangle and then repeatedly apply the verified procedure to count roots during the rectangle bisection phase. However, compared to modern complex procedures [4, 21], this simplistic approach suffers from several drawbacks:

- Our root counting procedure is based on remainder sequences, which are generally considered much slower than those built upon Descartes’ rule of signs.
- Modern isolation procedures are routinely required to deliver isolation boxes whose sizes meet some user-specified limit, hence they usually keep *refining* the isolation boxes even after the roots have been successfully isolated. The bisection strategy still works in the root refinement stage, but dedicated numerical approaches such as Newton’s iteration are commonly implemented for efficiency reasons.
- Modern isolation procedures sometimes prefer a bit-stream model in which the coefficients of the polynomial are approximated as a bit stream. This approach is particularly beneficial when the coefficients have extremely large bit-width or consist of algebraic numbers.
- Modern implementations usually incorporate numerous low-level optimisations, such as hash tables, which are hard to implement as verified procedures in a theorem prover.

Therefore, it is unlikely that our verified root counting procedures will ever deliver high performance. Nevertheless, they can be used to certify results from untrusted external root isolation programs, as in the certificate-based approach to solving univariate polynomial problems [13].

## Related Work

Formalisations of the winding number (from an analytical perspective) are available in Coq [3], HOL Light [7] and Isabelle/HOL. To the best of our knowledge, our tactic of evaluating winding numbers through Cauchy indices is novel. As both HOL Light and Isabelle/HOL have a relatively comprehensive library of complex analysis (i.e., at least including Cauchy’s integral theorem), our evaluation tactic could be useful when deriving analytical proofs in these two proof assistants.

The ability to count the *real* roots of a polynomial only requires Sturm’s theorem, so this capability is widely available among major proof assistants including PVS [18], Coq [15], HOL Light [17] and Isabelle [5, 10, 13]. However, as far as we know, our procedures to count *complex* roots are novel, as they require a formalisation of the argument principle [14], which is only available in Isabelle at the time of writing.

## Conclusion

In this paper, we have described a novel tactic  to evaluate winding numbers via Cauchy indices: given a goal of the form

$$\begin{aligned} n(\gamma _1+\gamma _2+\cdots +\gamma _n,z_0) = k, \end{aligned}$$

the tactic converts the target into an equality about Cauchy indices:

$$\begin{aligned} {\text {Indp}}(\gamma _1,z_0) + {\text {Indp}}(\gamma _2,z_0) + \cdots + {\text {Indp}}(\gamma _n,z_0) = -2 k. \end{aligned}$$

This can be then solved by individually evaluating ${\text {Indp}}(\gamma _1,z_0),\ldots ,{\text {Indp}}(\gamma _n,z_0)$. As open variables may occur in those Cauchy indices, the evaluation of them is unlikely to be fully automatic, but we provide lemmas (e.g., Lemma 11) to mitigate the laborious process. The tactic  has greatly helped us with the motivating proofs shown in Sect. 2, and we believe that it should be also beneficial in similar situations when dealing with winding numbers in a formal framework.

We have further related Cauchy indices to the argument principle and developed novel verified procedures to count the complex roots of a polynomial within the areas of rectangles and half-planes. Despite the limitations of not allowing roots on the border (which we will solve in future work), the ability to formally count complex roots is believed to lay the foundations for conducting stability analysis (e.g., the Routh–Hurwitz stability criterion) in the framework of the Isabelle theorem prover.

## Footnotes

1 Applying an introduction rule will replace a goal by a set of subgoals derived from the premises of the rule, provided the goal can be unified with the conclusion of the rule.
